# The Positive Effects of Physical Activity on Quality of Life in Parkinson’s Disease: A Systematic Review

**DOI:** 10.3390/geriatrics9040094

**Published:** 2024-07-15

**Authors:** Dharah P. C. F. Bispo, Carla C. S. A. Lins, Kelly L. Hawkes, Shae Tripp, Tien K. Khoo

**Affiliations:** 1 School of Medicine & Dentistry, Griffith University, Gold Coast, QLD 4222, Australia; 2 Neuropsychiatry and Behavioural Sciences Department, Health Sciences Centre, Federal University of Pernambuco, Recife 50670-901, PE, Brazil; 3 Gerontology Department, Health Sciences Centre, Federal University of Pernambuco, Recife 50670-901, PE, Brazil; 4 Anatomy Department, Health Sciences Centre, Federal University of Pernambuco, Recife 50670-901, PE, Brazil; 5 Northern New South Wales Local Health District, Ballina, NSW 2478, Australia; 6 Graduate School of Medicine, University of Wollongong, Wollongong, NSW 2522, Australia

**Keywords:** Parkinson’s disease, neurodegenerative diseases, exercise, physical activity, quality of life

## Abstract

Background: Physical activity can have positive effects on motor and non-motor symptoms in Parkinson’s disease, but its benefits in terms of quality of life and function are uncertain and vary based on the specific forms of activities and interventions. Objective: We sought to assess the current evidence on the positive effects of physical activity in people with Parkinson’s disease and more specifically in relation to its potential benefits for quality of life. Methods: This systematic review was conducted between January and April 2024 via the PubMed, Medline, and Scopus databases. Predetermined search criteria were used that included the following terms: “Parkinson’s disease”, “quality of life” and “physical activity”. Results: A total of 1669 articles were identified. After utilizing predetermined criteria, a total of fifteen articles met the selection criteria. Statistically significant improvements in quality of life were found in seven studies. Seven studies demonstrated a significant improvement in non-motor symptoms, while nine studies showed an improvement in motor symptoms. Conclusions: Despite heterogeneity in the study designs, interventions and clinical assessments, the articles identified in this review yielded mostly positive results in relation to physical activities. The findings reflect an improvement in motor and non-motor symptoms may translate to a better quality of life in people with Parkinson’s disease.

## 1. Introduction

Parkinson’s disease (PD) is a common neurodegenerative disorder typically characterized by motor symptoms and now increasingly recognized according to a myriad of complex non-motor symptoms (NMS). The latter are often subtle at onset and can occur many years prior to diagnosis [1,2,3,4]. With a wide array of symptomatology and the inevitable progression of the disease due to the loss of the dopaminergic neurons residing in the substantia nigra and non-dopaminergic dysfunction, quality of life (QoL) is predictably impacted in PD [5,6,7]. Early identification and treatment of symptoms can promote an improvement in the QoL of these individuals [8,9] and whilst management is often individualized as part of best practice, it can be challenging and ideally should encompass both pharmacological and non-pharmacological modalities [10,11].

When examining any purported treatment or procedure and its subsequent benefits or risks in terms of the outcomes for patients, QoL is an established construct used and universally understood to reflect the overall subjective well-being of an individual. It is not only pathophysiology and the manifestation of disease but also an individual’s function, satisfaction and contentment experienced in life which form the construct of QoL [12,13]. QoL outcomes are useful for attaining evidence of meaningful benefit, enhancing the significance of variables of interest and allowing for more holistic decision-making [12]. The link between QoL and physical activity (PA) has increasingly been consolidated in the literature [13], including in PD, whereby many studies have assessed various non-pharmacological interventions. Examples include exercise programs [14], Tai Chi Quan [15] and aquatic physiotherapy [16], which demonstrated an improvement in QoL and well-being in individuals with PD. PD-specific instruments have also been developed to assess QoL in this patient group [17,18,19,20,21].

Growing evidence indicates PA can offer tangible improvements in QoL measures for people with PD. The multisystem effects of PD require trials that examine PA and any reported benefits to account for its potential mechanisms, ranging from the simplistic approach of feeling more connected to others to its impact on neuroplasticity [22].

It is thought that exercise may complement standard pharmacological approaches by enhancing neuroplasticity in PD, such as regeneration and the survival of pars compacta neurons [23,24]. It is postulated that many neurotrophic factors are influenced by PA. For example, brain-derived neurotrophic factor (BDNF) is stimulated during exercise to mediate neuroprotective effects and is thought to improve cognition and mood [25,26,27]. Evidence suggests exercise can positively affect neuroplasticity via various mechanisms that include the up-titration of binding in the dopaminergic pathways [28,29] and inhibition of Lewy body formation in rat models [29]. Reducing neurodegeneration via the regulation of autophagy and apoptosis have also been proposed as mechanisms of the benefits of PA [25].

With feasible improvements in both, NMS (in terms of anxiety, cognitive functions and depression) and activities of daily living, exercise has the potential to significantly benefit the overall QoL in people with PD [30]. In this review, our defined objective was to assess the current evidence on the positive effects of physical activity in people with Parkinson’s disease and, more specifically, in relation to its potential benefit in terms of quality of life.

## 2. Materials and Methods

### 2.1. Literature Search and Selection Criteria

This systematic review was conducted between January and April 2024. The literature search used the guiding question of “Does physical activity positively affect quality of life in people with Parkinson’s disease?”. Based on a PICO approach, the population of interest comprised people with Parkinson’s disease with an interest in the positive effects of physical activity in the context of quality of life.

The terms in the present review, “Parkinson’s disease”, “quality of life” and “physical activity”, were used as search terms in three databases, PubMed, Medical Literature Analysis and Retrieval System Online (Medline) and Scopus, and were cross-checked with the use of Boolean AND. All terms are included in the Medical Subject Headings (MeSH) and Health Sciences Descriptors (DeCS).

**Figure 1:**
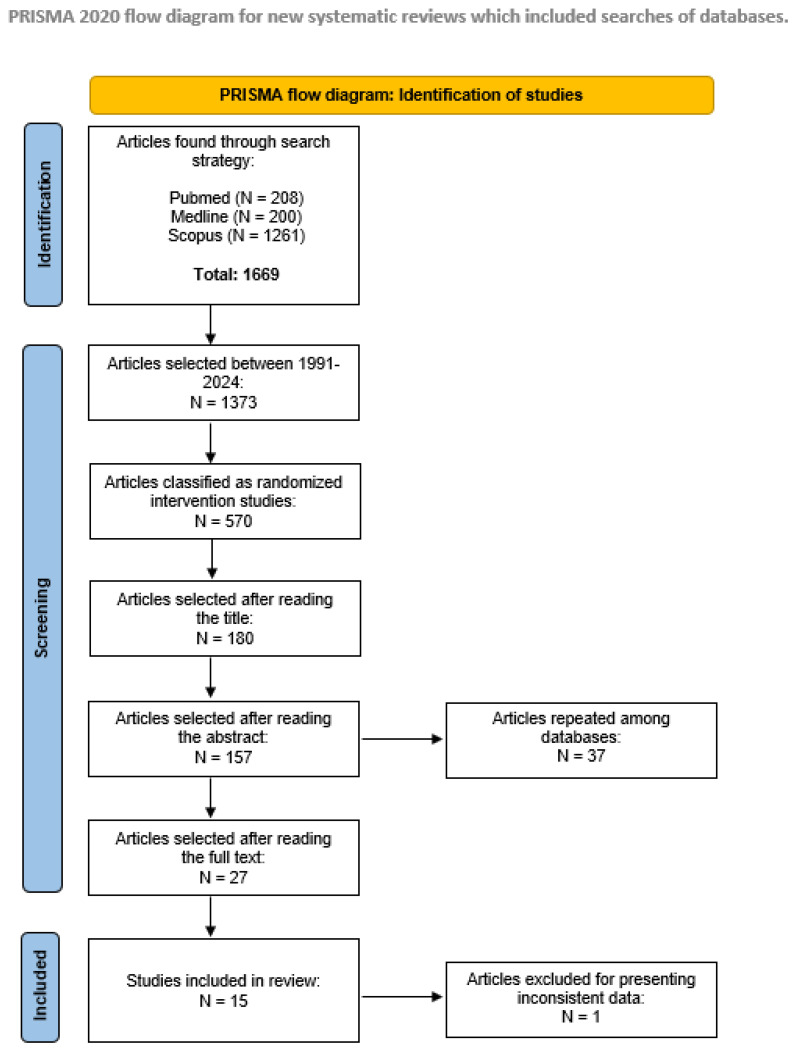
PRISMA 2020 systematic review flowchart of the selection of studies.

The inclusion criteria were as follows: (i) articles published between 1991 and 2024 written in English, Spanish or Portuguese; (ii) randomized interventional studies (level of evidence II) [31], which was specifically chosen to ensure the reliability and validity of the outcomes by minimizing bias; (iii) studies with 10 or more participants diagnosed with idiopathic PD in the intervention group, as well as appropriate evaluation of the effects of physical activity and quality of life. The exclusion criteria comprised the following: (i) studies in animals, letters to the editor or systematic or integrative reviews and (ii) repeated articles in different databases. The different phases of the systematic review are summarized in Figure 1.

Articles were blindly and independently selected by two reviewers, after which the abstracts were read. After that, two more reviewers were added, and the JADAD scale was applied. The JADAD consists of 5 questions that assess the following aspects of clinical trials: randomization, blinding and description of losses to follow-up [32]. These measures ensured the reliability and validity of the studies in question.

The protocol for this systematic review was registered with PROSPERO on 25 October 2021. 

### 2.2. Outcomes of Interest

The primary outcomes related to QoL were assessed using the following instruments: Parkinson’s disease questionnaire-8 (PDQ-8), Parkinson’s disease questionnaire-39 (PDQ-39), Parkinson’s disease Quality of Life Questionnaire (PDQL), the 33-item Parkinson’s disease quality of life questionnaire (PDQUALIF), EuroQoL five-dimension (EQ-5D) and the Short Form Health Survey (SF-36).

As QoL has a global scope, its associated effects on motor symptoms and NMS were considered as secondary outcomes. A diverse array of instruments for assessing the latter were used based on the respective study objectives and variables of interest. Therefore, the following instruments were utilized:

- Assessment of motor symptoms: Two-Minute Walk Test (2MWT), 6-Minute Walk Test (6MWT), Berg Balance Scale (BBS); Continuous-Scale Physical Functional Performance Test (CS-PFP), Falls Efficacy Scale International (FES-I), Freezing of Gait Questionnaire (FOG, Functional Reach Test (FRT), Physical Activity Questionnaire (IPAQ), Mini-Balance Evaluation Systems Test (MBEST), Movement Disorder Society Unified Parkinson’s Disease Rating Scale (MDS-UPDRS), Sit-to-Stand Test (STS), Test of Attentional Performance Flexibility (TAPF), Timed Up and Go (TUG).
- Assessment of non-motor symptoms: Fear of Falling Avoidance Behavior Questionnaire (FFABQ), Parkinson’s Disease Non-Motor Symptom Questionnaire (N-MSQ), Parkinson’s Disease Sleep Scale (PDSS), Scales for Outcomes in Parkinson’s Disease (SCOPA)—sleep and gastrointestinal, Parkinson Fatigue Scale (PFS).
- Assessment of affective symptoms: Beck Depression Inventory (BDI), Fatigue Severity Scale (FSS), Geriatric Depression Scale (GDS), Hospital Anxiety and Depression Scale (HADS), State-Trait Anxiety Inventory (STAI).
- Cognitive assessment: Mini Mental State Examination (MMSE), Montreal Cognitive Assessment (MoCA), Test of Attentional Performance (TAP), Trail Making Test (TMT).

### 2.3. Data Extraction 

Data extraction was conducted by one author and confirmed by the co-authors. After applying the inclusion and exclusion criteria, each article was reviewed in detail by two nominated members, with particular interest in the participant demographics, type of PA, frequency, duration and mode of delivery, as well as the effect on QoL.

## 3. Results

The selection process identified 1669 articles, of which 980 were excluded due to lack of randomization or because they had not been published between 1991 and 2024. A further 502 articles were excluded following title and abstract screening. The remaining 150 articles were reviewed and resulted in 14 articles meeting the inclusion criteria (see Figure 1—flow diagram). 

The selected articles were in their entirety randomized interventional studies published between 2012 and 2024. With respect to the location of the respective studies, five studies were from North America [33,34,35,36,37]; two were from South America: Brazil [38,39]; five were from Europe: Hungary [40], Italy [41], the United Kingdom [42], Germany [43]; The Netherlands [44]; and three were from Asia: the Republic of Korea [30] and Hong Kong [45,46]. All of the articles were published in English.

### 3.1. Participants

The number of participants in each study ranged from 20 to 230, with an overall total of 1200 participants (see Table 1). There was a higher male-to-female ratio in all studies apart from three, which had a higher female-to-male ratio [30,45,46]. The duration of PD since diagnosis ranged from 1 to 15 years in the selected sample, with the exception of two studies that reported this information at baseline but did not specify disease duration [45,46]. Though duration of disease was considered as a variable, the researchers did not present these data in the latter study.

### 3.2. Medication

Two studies did not mention whether the participants were analyzed in an ON or OFF state [30,40,43]. Three studies did not detail medication use [30,39,43]. The use of dopaminergic medications (levodopa and/or dopamine agonist) was defined as the inclusion criterion for only one study [44].

In contrast, seven studies assessed participants in the ON state [34,35,36,37,38,39,45]. Only two studies assessed participants in both the ON and OFF states [33,44].

The mean levodopa-equivalent daily dose (LEDD) with a standard deviation (range) was presented in only four studies, these being 725.0 ± 234 mg/day (PD Irish dance) and 645.0 ± 216 mg/day (control) [41], 843.4 ± 308.8 mg/day (high-intensity agility program) and 884.8 ± 332.0 mg/day (control group) [40], 419.3 ± 389.2 mg/day (high-intensity multimodal exercise boot camp) and 476.7 ± 300.0 mg/day (control) [33] and 766.4 ± 607.2 (mindfulness meditation) and 518.5 ± 562.3 mg/day (control) [45]. Two studies detailed the various types of levodopa replacement therapy utilized [38,41].

### 3.3. Intervention and Activity Type

A variety of interventions were used, including a high-intensity multimodal boot camp [33], yoga [34,35,46], aerobic exercise [37,40,44], flexibility and function training [37], stretching and resistance training exercises [39,45,46], mindfulness meditation-based exercise [30,45], high-intensity agility training [40], Brazilian dance [38], deep-water exercise [38], Nordic walking [38,41], Irish dancing [41], physiotherapy exercise [41,43], multimodal Parkinson’s complex treatment [43], gym-based exercises [45], free weight exercise [39] and power weight training [36] (see Table 2). The duration of the intervention ranged from 3 weeks to 16 months. The frequency of the intervention varied from 1 to 5 sessions per week and encompassed a minimum of 30 min and a maximum of 120 min per session. Two studies had a relatively short intervention duration of between 3 and 8 weeks [33,40,46], whilst seven studies had a median duration of 3 to 9 months [34,35,36,41,42,43,44]. One study had a longer duration of 16 months [37].

Different professionals were involved in the facilitation of PA in the respective studies, and as such, the nature of the PA varied accordingly. Several professionals were involved in delivering and facilitating PA as an intervention, such as physiotherapists, personal trainers, dance teachers and yoga teachers/instructors.

### 3.4. Measurement Tools

The most commonly used instrument to assess QoL was the 39-item Parkinson’s Disease Questionnaire (PDQ-39), which was utilized in seven studies [33,35,38,39,40,41,44] (see Table 3).

A variety of measures and instruments were used to evaluate motor symptoms. Seven studies used the Movement Disorder Society—Unified Parkinson’s Disease Rating Scale (MDS-UPDRS) [33,38,40,42,44,45,46], and five studies used the UPDRS [34,36,37,39,41]. Most of the studies utilized part III of the MDS-UPDRS or the UPDRS for motor assessment [33,34,36,37,38,39,41,42,44,45,46]. Only one study used part II (motor aspects of experience of daily living) [40] and IV (motor complications—dyskinesia and fluctuation) of the MDS-UPDRS [44]. One study used the UPDRS total score [37]. Two studies did not use the UPDRS [30,43].

In terms of non-motor symptom assessment, the studies identified utilized various instruments, with only one study using the Non-Motor Symptoms Questionnaire (N-MSQ) [42]. The non-motor symptom assessments are further summarized in Table 4, and one of the fourteen studies did not evaluate any NMS [35]. In addition, part I of the UPDRS was used to assess the non-motor aspects of experiences of daily living in only one study [37].

### 3.5. Methodology Quality

Based on the level of evidence and selection criteria, all 15 studies comprised randomized clinical trials (Cochrane Level of Evidence II). Nine studies had a single-blinded component [33,34,37,39,40,41,42,45,46], whilst one study was double-blinded [44]. The other five studies had no blinding [30,35,36,38,43]. In the majority of studies, the control group involved a smaller active component of home-based exercise. 

### 3.6. Positive Effects on Outcomes of Interest

Statistically significant improvements in QoL were found in seven studies using the EQ-5D, PDQL, PDQ-8 and PDQ-39 s [30,35,36,39,40,43,46]. The remainder of the articles either had no statistically significant improvement in QoL or did not specify *p*-values for analysis. Seven studies demonstrated a statistically significant improvement in NMS [30,33,38,40,43,45,46], whilst nine studies showed an improvement in motor symptoms [30,33,38,39,40,41,44,45,46].

Analysis of the type of intervention performed in the studies showed significant benefits in the QoL scores for yoga practice [35,46] and the Mindfulness Mediation-Based Complex Exercise Program (MMBCEP) [30]. Another study that applied PWT as a different form of exercise also had a positive impact on QoL [36]. The study by Son et al. (2018) included stretching and complex strength exercises [30]. As such, it could also be compared to the PA intervention performed in the previous study [36]. The frequency and duration varied among studies.

Meditation performed as part of the intervention in one study focused on different subjects, such as respiration, loving and imagery training. This study demonstrated effects not only on physical aspects but also psychological/mental aspects with a reduction in negative self-images of oneself [30]. A similar approach of mind-body exercises was also adopted by Kwok et al. [46]. Yoga showed QoL improvements in the mobility domain and the overall PDQ-39 score [35,46]. Some interventions focused on specific aspects of motor function such as muscular endurance [30]; general strength [33]; upper extremity muscular strength [30]; lower extremity muscular strength [35]; balance [30,41,44]; mobility [41,44]; gait [33,34,41,44]; bradykinesia [33,34,41,44]; posture [33,34,41,44] as well as reaching and grasping [41]. Studies that yielded positive effects on QoL and NMS assessed depression, anxiety, cognitive function [30] and sleep disturbance [30]. Studies by Ni et al. also showed positive effects on QoL, although these studies did not evaluate NMS [35,36].

Two studies measured PA with the 31-item Longitudinal Aging Study Amsterdam Physical Activity Questionnaire (LAPAQ) [34,44], whilst one study each used the International Physical Activity Questionnaire (IPAQ) [33] and the Physical Activity Scale for the Elderly [42], respectively. 

In terms of activities of daily living and QoL, meaningful results were shown in two studies that utilized differing instruments, namely the Activities of Daily Living scale, the Schwab and England Activities of Daily Living Scale and MDS-UPDRS-ADL part II [40,41]. In addition, falls were evaluated in two studies via questionnaires and considered as complications that can compromise motor function [33,44].

## 4. Discussion

### 4.1. Physical Activity in Parkinson’s Disease and Its Impact in Quality of Life

Adherence to PA may be perceived as challenging for individuals affected by neurodegenerative diseases such as PD, whereby the symptom burden increases with disease progression [47]. The barriers to initiating and maintaining regular exercise routines are multifaceted, including (1) body structure and function, of which PD motor and non-motor symptoms are part, (2) activities and participation, (3) personal and (4) environmental. Factors such as advancing age, comorbidities and frailty, alongside varying responses to treatment, can impede adherence to PA regimes. The detriment of reduced PA and a lack of mobility are postulated to accelerate frailty, fall risks, immobility and reduced QoL. When combined, a lack of PA may contribute towards an increased risk of hospitalization and the need for long-term care.

This complex interplay between physical activity and health outcomes necessitates a robust method for evaluating its effect on QoL, whereby the latter is considered a broad concept that encompasses biopsychosocial and spiritual well-being and should not be solely considered an absence of disease [48]. In assessing QoL, homogeneity was observed across studies that utilized the PDQ-39. 

Enhancements in QoL across different PA modalities that comprise individual and group forms of intervention, as well as facility- and home-based programs, highlight the potential of tailored physical activities to mitigate the barriers to implementation with the intention of improving overall health outcomes. In this review, we found one study that involved individual exercise programs at designated facilities and home-based regimes had yielded positive results in the outcomes measured, which included improvements in daily living activities and social support [36].

### 4.2. Motor Symptom Benefit

Physical therapy and specialized exercise programs have shown significant benefits in PD. A sensorimotor agility boot camp, involving activities like Tai Chi, boxing, lunges, kayaking, agility courses and Pilates, notably improved gait measures [33]. Irish dance, although not initially sought after by individuals with PD, presents itself as a strategy that can improve mobility, thereby contributing to an enhanced quality of life [41]. Additionally, it is an activity that can also be enjoyable and performed together with other family members. Conversely, the control group, engaging in a physiotherapy program, reported less benefits.

Resistance exercises and power resistance training (PWT) were particularly effective, showing more significant improvements in mobility [40,42] and muscle strength [30,36], as well as upper and lower limb bradykinesia [36].Furthermore, high-intensity exercise programs in non-demented individuals with mild to moderate stage PD may prove to be beneficial in terms of mobility and balance, thus facilitating the maintenance of independence and functional well-being [40]. Moreover, improvements were also noted in areas related to endurance, coordination, agility, and balance [30,40], which further supports the role of varied and targeted exercise regimens in the management of PD.

Benefits related to dance therapy were seen in gains in the “Timed Up and Go test” and reductions in the Freezing of Gait Questionnaire scores given that rapid movements and step routines that are crucial for minimizing motor symptoms and enhancing balance and flexibility, potentially increasing the independence of individuals [41].

### 4.3. Non-Motor Symptom Benefits

It has been postulated that individuals in the early stages of PD, particularly those who retain cognitive abilities, may derive more significant benefits from PA. This advantage is likely due to the dependency of such activities on executive functions, which include attention and processing speed [49]. 

The effectiveness of home exercise programs was thought to be notably influenced by effect modifiers such as depression and cognitive impairment; importantly, age did not contribute significantly to the study findings [50]. A study that compared a high-intensity multimodal exercise boot camp with the usual care found that the former significantly enhanced intrinsic motivation [33]. Additionally, adherence to regular physical activity not only reduced fatigue [47] but also provided broader neurological health benefits, thereby improving overall quality of life [39].

Incorporating mindfulness meditation into complex exercise routines demonstrated substantial benefits. These activities enhance cognitive function and emotional well-being, which is thought to be highly relevant in the context of non-motor symptoms [45]. The effectiveness of combining mindfulness-based stress reduction practices with PA, particularly in managing non-motor symptoms such as depression and anxiety, has also been evaluated positively [30,46]. This combined approach has led to reduced anxiety and improvements in concentration, memory and performance in ADLs.

### 4.4. Benefits of Integrative and Synergistic Therapy

While traditional PA has long been validated within therapeutic contexts, emerging evidence underscores the efficacy of integrative therapies such as meditation and yoga. Studieshave demonstrated substantial improvements in depressive symptoms, mindfulness and cognitive performance among participants engaged in these practices [45,46]. Additionally, these modalities have been shown to enhance psychospiritual outcomes, which directly mitigate symptoms of depression and anxiety [30,45].

Furthermore, the impact of such therapies extends to motor function improvements. Son et al. 2018 observed significant enhancements in physical performance measures, including the chair stand test, shoulder flexibility and walking tests such as the Six-Minute Walk Test [30]. Meditation has been associated with increased joint flexibility, a decrease in resting tremor and general improvements in motor muscle function [30,34]. These improvements are crucial, as they directly enhance performance in daily activities and the quality of life of both participants and their carers.

A significant decrease in bradykinesia scores and stiffness in both the upper and lower limbs following yoga interventions again suggests that an integrative and synergistic approach to physical activity interventions in PD may help maximize its therapeutic benefits [35].

### 4.5. Potential Therapeutic Mechanisms

An increase in BDNF following PA has been relatively well investigated by numerous studies that suggest its role in epigenetic processes that contribute towards synaptic neuroplasticity [51,52,53]. Furthermore, exercise and its effects on cardiovascular-related microRNAs are likely to result in broader beneficial neurovascular effects which may contribute towards neuronal health [54,55]. The expression levels of specific microRNAs have also been described in PD-specific studies that demonstrated exercise was associated with an improvement in cognition [56]. The neurophysiological effects of exercise are further elucidated by studies that support changes in and the modulation of neural networks and oscillations, as reflected by functional neuroimaging (such as the upregulation of resting state networks) and electroencephalography, thus highlighting the state of local and inter-regional neural synchrony as crucial to appreciate in relation to an individual’s function in the setting of neurodegenerative diseases such as PD [57,58,59]. In addition, growing interest of the glymphatic system in PD and its beneficial modulatory effects on protein clearance and cerebrovascular indicators further strengthens the far-reaching effects of PA on brain health [60,61,62,63].

### 4.6. Limitations

The articles identified in this review indicate various forms of PA may be beneficial; however, comparison between the studies is challenging due to the differences in the PA interventions and overall study heterogeneity. Studies of this nature are often subject to a proportion of selection and participation bias, as well as potential Hawthorne effects. Another challenge perceived by the researchers was the performance of PA in “non-exercisers”, which may be subject to the latter factors.

## 5. Conclusions

Despite differences in the study interventions and constructs, studies to date on the effects of PA on PD suggest tangible benefits in terms of both motor symptoms and NMS. Although a consensus recommendation on the best form of exercise as an intervention in PD is not currently available, it is likely that PA will be beneficial with a risk of minimal harm if patients are selected appropriately and the intervention is conducted in a safe environment. The positive impact of PA on QoL may be more significant in individuals who are able to consistently adhere and engage. Individualized PA interventions may provide better outcomes and are likely required with disease progression. Further research on PA is required to determine the best forms of therapy in people with PD and across the spectrum of its symptom burden. This would be further supported by studies that indicate the positive association of PA and neural function with the strong potential of this therapeutic modality to be better translated to and applied in the management of PD.

## Figures and Tables

**Table 1:** Cohort demographics, including intervention and control groups.

Study	Country	Total Number of Participants	Groups		Participants	Mean Age (Years) (SD)	Gender (M:F)	Duration of PD (Years) (SD)	Dropouts
Haas et al., 2023 [38]	Brazil	83	InT	BD	37	71.61 (8.89)	10:21	5.6 (5.09)	6
				DWE	22	66.76 (8.97)	17:4	8.0 (4.65)	1
				NW	35	67.87 (11.2)	23:8	7.0 (5.07)	4
Kwok et al., 2023 [45]	Hong Kong	68	InT-MM		33	62.7 (7.7)	10:23	N/A	2
			Control-SRTE		35	66.1 (8.9)	19:16	N/A	1
Wagner et al., 2022 [43]	Germany	230	InT-PTP		93	64.1 (9.3)	62:30	7.75 (6.2)	15
			Control-MKP		137	67.6 (9.3)	84:51	8.23 (5.1)	8
Chen et al., 2021 [39]	Brazil	74	InT	GG	23	63.4 (6.9)	17:6	7.6 (6)	2
				FG	26	63.2 (6.4)	18:8	8.4 (5.9)	6
			Control-SE		25	63.6 (7)	18:7	N/A	4
Landers et al., 2019 [33]	USA	27	InT-HIBC		14	63.5 (10.9)	10:4	4.9 (5.1)	1
			Control-UC		13	64.6 (6.0)	9:4	4.7 (3.9)	2
Kwok et al., 2019 [46]	Hong Kong	138	Int-YP		71	63.7 (8.2)	37:34	N/A	14
			Control-SRTE		67	63.5 (9.3)	28:39	N/A	8
van der Kolk et al., 2019 [44]	The Netherlands	130	Int-AIG		65	59.3 (8.3)	42:23	41 months (16–87)	4
			Control-ACG		65	59.4 (9.3)	38:27	38 months (19–81)	1
Cheung et al., 2018 [34]	USA	20	InT-HY		10	63.5 (8.5)	N/A	1–5 yrs = 7 6–10 yrs = 2 11–15 yrs = 1	0
			Control-WL		10	65.8 (6.6)	N/A	1–5 yrs = 4 6–10 yrs = 6 11–15 yrs = 0	1
Son et al., 2018 [30]	Republic of Korea	63	InT-MMBCEP		33	NA	14:19	<3 yrs = 21 >3 yrs = 12	0
			Control-ROTP		30	NA	9:21	<3 yrs = 24 >3 yrs = 6	3
Tollár et al., 2018 [40]	Hungary	64	InT-HIAP		35	67.2 (3.4)	17:18	6.7 (2.3)	0
			Control-NPICG		29	67.6 (4.1)	12:8	7.1 (2.8)	9
Collett et al., 2016 [42]	United Kingdom	105	InT-EG		54	66 (9)	31:23	4.8 (4.1)	17
			Control-HG		51	67 (7)	30:21	5.3 (4.1)	11
Ni et al., 2016 [35]	USA	27	InT-YP		15	71.2 (6.5)	11:4	6.9 (6.3)	2
			Control-UC		12	74.9 (8.3)	6:6	5.9 (6.2)	2
Ni et al., 2016 [36]	USA	26	InT-PWT		14	71.6 (6.6)	9:5	6.6 (4.4)	0
			Control-CON		12	74.9 (8.3)	4:6	5.9 (6.2)	2
Volpe et al., 2013 [41]	Italy	24	InT-ID		12	61.6 (4.5)	7:5	9.0 (3.6)	N/A
			Control-PT		12	65.0 (5.3)	6:6	8.9 (2.5)	N/A
Schenkman et al., 2012 [37]	USA	121	InT-FBF		39	64.5 (10.0)	24:15	4.9 (3.7)	6
			InT-AE		41	63.4 (11.2)	26:15	3.9 (4.2)	10
			Control-HEG		41	66.3 (10.1)	26:15	4.5 (3.8)	9

ACG: active control group (stretching, flexibility and relaxation exercises); AE: supervised aerobic exercise; AIG: aerobic intervention group; BD: Brazilian dance; CON: non-exercise control group; DWE: deep-water exercise; EG: exercise group; F: female; FBF: supervised flexibility/balance/function exercise; FG: free weight and elastic band group; GG: gym group; HEG: home exercise group; HIAP: high-intensity agility program; HIBC: high-intensity multimodal exercise boot camp; HG: handwriting group; HY: Hatha yoga; ID: Irish dance; InT: intervention; M: male; mins: minutes; MKP: multimodal Parkinson’s complex treatment; MM: mindfulness meditation; MMBCEP: Mindfulness Meditation-Based Complex Exercise Program; N/A: not available; NPICG: no physical intervention control group; NW: Nordic walking; PTP: physiotherapy training program; PWT: power-based resistance training; ROTP: routine outpatient therapeutic program; SD: standard deviation; SE: stretching exercises SRTE: stretching and resistance training exercise; UC: usual care; WL: waitlist control group; YP: yoga program.

**Table 2:** Interventions and methods of identified studies.

Study	Intervention	Frequency	Length of Session (Minutes)	Duration of Intervention	JADAD Score
Haas et al., 2023 [38]	BD vs. DWE vs. NW	24 sessions	BD: 55–65 DWE: 60 NW: 60	3 months	4
Kwok et al., 2023 [45]	MM vs. SRTE	1 session per week	MM: 90 SRTE: 90	2 months	4
Wagner et al., 2022 [43]	PTP vs. MKP	Up to 3 sessions per week	NA	9 months	4
Chen et al., 2021 [39]	GG vs. FG vs. Control	2 sessions per week	GG: 50 FG: 50 Control: 50	3 + 6 months	3
Landers et al., 2019 [33]	HIBC vs. Control	3 sessions per week	HIBC: 90 UC: 60	2 months	5
Kwok et al., 2019 [46]	YP vs Control	1 session per week	YP: 90 Control: 60	2 months	3
van der Kolk et al., 2019 [44]	AIG vs. Control	3 sessions per week	AE: 30–45 Control: 30	6 months	5
Cheung et al., 2018 [34]	HY vs. Control	2 sessions per week	HY: 60 Control: NI	3 months	3
Son et al., 2018 [30]	MMBCEP vs. Control	6 sessions in total	MMBCP: 120 Control: NI	2 months	3
Tollár et al., 2018 [40]	HIAP vs. Control	15 sessions over 3 weeks	HIAP: 60 Control: NI	>1 months	3
Collett et al., 2016 [40]	EG vs. Control	2 sessions per week	Exercise: 60 Control: 60	6 months	5
Ni et al., 2016 [35]	YP vs. Control	2 sessions per week	YP: 60 Control: NDT	3 months	3
Ni et al., 2016 [36]	PWT vs. Control	2 sessions per week	PWT: NDT Control: 60	3 months	3
Volpe et al., 2013 [41]	SPG vs. ID	1 session per week	SPG: 90 ID: 90	1 + ½ month	2
Schenkman et al., 2012 [37]	FBF vs. AE vs. Control	FBF and AE: 3 sessions per week for 4 months, then once a month for rest of study Control: once a month	AE: 40–50 FBF: NDT Control: NDT Individual sessions: NDT for any intervention	16 months	5

AE: supervised aerobic exercise; AIG: aerobic intervention group; BD: Brazilian dance; DWE: deep-water exercise; EG; exercise group; FBF: supervised flexibility/balance/function exercise; FG: free weight and elastic band group; GG: gym group; HIAP: high-intensity agility program; HIBC: high-intensity multimodal exercise boot camp; HY: Hatha yoga; ID: Irish dance group; mins: minutes; MKP: multimodal Parkinson’s complex treatment; MM: mindfulness meditation; MMBCEP: Mindfulness Meditation-Based Complex Exercise Program; NDT: no defined time; NI: no intervention; NW: Nordic walking; PTP: physiotherapy training program; PWT: power-based resistance training; SPG: standard physiotherapy group; SRTE: stretching and resistance training exercise; YP: yoga program.

**Table 3:** Effects of physical activities on quality of life and functional well-being.

Study	Instruments	Variable	Pre-Intervention Mean (SD)	Post-Intervention Mean (SD)	*p*-Value
Haas et al., 2023 [38]	PDQ-39	BD	34.35 (3.21) *	32.97 (2.16) *	0.12
		DWE	34.90 (3.52) *	35.76 (3.39) *	
		NW	31.87 (2.79) *	27.77 (2.25) *	
Kwok et al., 2023 [45]	PDQ-8	MM	NA	36.26 (17.58)	0.287
		SRTE	NA	28.17 (15.45)	0.547
Wagner et al., 2022 [43]	PDQ-8	PTP	26.8	27.5	<0.01
		MKP	29.8	33.0	
Chen et al., 2021 [39]	PDQ-39—Mobility	GG	34.72 (24.10)	25.8 (25.16)	0.019
		FG	30.52 (22.02)	24.63 (20.63)	
		Control	23.12 (19.51)	23.52 (17.97)	
Landers et al., 2019 [33]	PDQ-39	HIBC	NA	21.1 ± 5.5	0.328
		Control	NA	21.7 ± 5.5	0.484
Kwok et al., 2019 [46]	PDQ-8	YP	9.8 (5.0)	6.0 (4.8)	<0.001
		Control	9.2 (5.3)	8.8 (5.5)	
van der Kolk et al., 2019 [44]	PDQ-39	AIG	24.9 (2.2) *	26.0 (2.3) *	0.910
		Control	24.0 (2.2) *	26.3 (2.3) *	
Cheung et al., 2018 [34]	PDQUALIF	HY	55 (15.8)	55 (15.8)	NA
		Control	60 (17.5)	50 (23.6)	
Son et al., 2018 [30]	PDQL	MMBCEP	136.27 (30.45)	153.63 (21.66)	0.006
		Control	147.83 (24.77)	139.27 (17.84)	
Tollár et al., 2018 [40]	PDQ-39	HIAP	30.0 (8.3)	23.4 (7.2)	<0.001
		Control	30.6 (15.0)	30.8 (13.8)	
	EQ-5D	HIAP	0.5 (0.1)	0.5 (0.1)	<0.05
		Control	0.4 (0.2)	0.5 (0.1)	
Collett et al., 2016 [42]	EQ-5D	EG	76 (15.4)	76 (2)	0.903
		Control	75 (15)	62 (3)	
	SF-36	Physical EG	64 (18)	68 (3)	0.397
		Physical Control	61 (19)	74 (2)	
		Mental EG	71 (17)	58 (3)	0.470
		Mental Control	68 (17)	66 (3)	
Ni et al., 2016 [35]	PDQ-39	YP	44.2 (32.5)	NA	0.016
		Control	34.2 (16.9)	NA	
Ni et al., 2016 [36]	PDQ-39	PWT	39.3 (13.4)	NA	0.028
		Control	35.2 (20.4)	NA	
Volpe et al., 2013 [41]	PDQ-39	ID	30.60 (12.06)	22.16 (10.18)	0.153
		Control	32.58 (7.59)	27.61 (7.67)	
Schenkman et al., 2012 [37]	PDQ-39	FBF	23.2 (13.6)	17.2 (2.1)	0.64
		AE	18.5 (13)	17.1 (2.3)	
		Control	21.5 (9.6)	21.0 (2.2)	

AE: supervised aerobic exercise; AIG: aerobic intervention group; BD: Brazilian dance; DWE: deep-water exercise; EG: exercise group; EQ-5D: EuroQoL five-dimension; FBF: flexibility/balance/function exercise; FG: free weight and elastic band group; GG: gym group; HIAP: high-intensity agility program; HIBC: high-intensity multimodal exercise boot camp; ID: Irish dance; MKP: multimodal Parkinson’s complex treatment; MM: mindfulness meditation; MMBCEP: Mindfulness Meditation-Based Complex Exercise Program; NA: not available; NW: Nordic walking; PDQ-39: Parkinson’s Disease Questionnaire-39; PDQ-8: Parkinson’s Disease Questionnaire-8; PDQL: Parkinson’s Disease Quality of Life Questionnaire; PDQUALIF: 33-item Parkinson’s Disease Quality of Life Questionnaire; PTP: physiotherapy training program; PWT: power-based resistance training; SF-36: Short Form Health Survey; SRTE: stretching and resistance training exercise; YP: yoga program. * Mean (SE).

**Table 4:** Effects of physical activities on motor and non-motor symptoms.

Study	Instruments		Group	Pre-Intervention Mean (SD)	Post-Intervention Mean (SD)	*p*-Value
Haas et al., 2023 [38]	Motor symptoms	MDS-UPDRS III	BD		14.16 (1.24)	0.78
			DWE	16.09 (1.19)	17.62 (1.46)	
			NW	14.16 (0.99)	14.65 (1.18)	
		FES-I	BD	32.00 (1.62)	30.35 (1.47)	0.98
			DWE	29.76 (1.89)	31.95 (2.23)	
			NW	30.00 (1.69)	32.13 (1.83)	
		STS	BD	21.53 (1.76)	14.04 (0.81)	0.89
			DWE	18.04 (1.77)	16.77 (1.79)	
			NW	16.25 (0.86)	17.34 (1.86)	
		TUG-SSS	BD	14.93 (1.18)	11.29 (0.66)	0.76
			DWE	12.22 (0.79)	12.68 (1.89)	
			NW	13.56 (1.18)	13.26 (1.10)	
		TUG-FS	BD	11.70 (0.97)	9.06 (0.51)	0.58
			DWE	9.38 (0.53)	9.62 (1.23)	
			NW	9.72 (0.59)	9.7 (0.94)	
		6MWT	BD	428.71 (22.81)	434.14 (16.2)	0.14
			DWE	455.95 (19.22)	478.32 (22.58)	
			NW	452.13 (17.73)	477.91 (17.87)	
		Handgrip test	BD	43.77 (2.52)	52.93 (3.81)	0.01
			DWE	57.14 (4.63)	56.33 (3.82)	
			NW	61.10 (3.62)	59.86 (2.72)	
	Non-motor symptoms	MoCA	BD	21.94 (0.95)	22.35 (0.83)	0.06
			DWE	22.81 (1.33)	24.4 (0.88)	
			NW	24.27 (0.69)	24.06 (0.86)	
Kwok et al., 2023 [45]	Motor symptoms	MDS-UPDRS III	MM	NA	32.51 (11.44)	0.09
			SRTE	NA	32.36 (11.91)	0.01
		TUG	MM	NA	12.61 (6.63)	0.21
			SRTE	NA	12.28 (3.83)	0.12
	Non-motor symptoms	HADS—Anxiety	MM	NA	6.66 (3.80)	0.60
			SRTE	NA	6.28 (3.82)	0.10
		HADS—Depression	MM	NA	6.91 (3.26)	0.11
			SRTE	NA	6.29 (3.45)	0.71
		MoCA—Visuospatial	MM	NA	4.11 (1.01)	0.20
			SRTE	NA	4.30 (1.21)	0.42
		MoCA—Naming	MM	NA	2.85 (0.37)	1.00
			SRTE	NA	2.85 (0.50)	0.57
		MoCA—Attention	MM	NA	5.45 (0.64)	0.44
			SRTE	NA	5.52 (0.63)	0.28
		MoCA—Language	MM	NA	2.80 (0.41)	0.66
			SRTE	NA	2.83 (0.46)	0.32
		MoCA—Abstraction	MM	NA	1.43 (0.58)	1.00
			SRTE	NA	1.34 (0.60)	0.37
		MoCA—Delayed Recall	MM	NA	4.10 (1.30)	0.04
			SRTE	NA	3.77 (1.25)	0.01
		MoCA—Orientation	MM	NA	5.96 (0.19)	0.10
			SRTE	NA	5.88 (0.42)	0.04
		MoCA—Total	MM	NA	26.77 (2.61)	0.01
			SRTE	NA	26.70 (3.33)	0.01
Wagner et al., 2022 [43]	Motor symptoms	NA				
	Non-motor symptoms	PDSS	PTP	17.8	18.6	<0.01
			Control	19.5	20.8	
Chen et al., 2021 [39]	Motor symptoms	UPDRS III	GG	29.13 (10.06)	27.65 (9.92)	0.01
			FG	29.58 (12.06)	28.38 (10.05)	
			Control	26.44 (9.95)	27.60 (8.12)	
		TUG	GG	8.70 (3.39)	7.91 (2.89)	0.89
			FG	8.5 (2.10)	7.96 (1.93)	
			Control	8.56 (1.73)	8.12 (1.88)	
		BBS	GG	52.09 (4.5)	52.96 (2.93)	0.04
			FG	51.00 (4.74)	52.96 (2.82)	
			Control	52.28 (2.79)	52.24 (3.07)	
		MBEST	GG	24.48 (4.24)	25.70 (4.24)	0.01
			FG	23.69 (4.71)	25.69 (3.92)	
			Control	24.92 (4.14)	25.04 (3.66)	
	Non-motor symptoms	MMSE	GG	NA	27.4 (1.9)	0.53
			FG	NA	26.9 (2.4)	
			Control	NA	27.5 (2.1)	
Landers et al., 2019 [33]	Motor symptoms	IPAQ	Vigorous HIBC	77.8 (35.9)	168.9 (116.3)	1.00
			Vigorous Control	33.3 (20.9)	86.7 (35.2)	0.03
			Moderate HIBC	86.1 (49.6)	236.1 (127.2)	0.004
			Moderate Control	85.6 (44.8)	245.6 (195.9)	0.22
			Walk HIBC	135.6 (42.1)	333.9 (193.8)	0.45
			Walk Control	312.2 (66.2)	217.8 (80.9)	0.07
			Sit HIBC	507.8 (81.2)	453.3 (56.7)	0.73
			Sit Control	363.4 (76.5)	393.3 (97.1)	0.75
		MDS-UPDRS III	On HIBC	25.8 (4.7)	16.3 (4.8)	0.02
			On Control	35.6 (4.4)	24.2 (4.5)	0.05
			Off HIBC	32.3 (5.0)	23.6 (4.5)	0.05
			Off Control	36.5 (5.3)	35.0 (4.8)	0.11
		FFABQ	HIBC	8.9 (4.3)	8.5 (4.0)	0.15
			Control	12.3 (4.0)	11.6 (3.8)	0.62
		6MWT	On HIBC	491.9 (32.5)	515.1 (39.7)	0.02
			On Control	418.3 (48.8)	440.4 (68.5)	0.01
			Off HIBC	456.7 (42.2)	484.6 (46.4)	0.05
			Off Control	394.6 (45.1)	442.7 (49.6)	0.09
		STS	On HIBC	11.5 (2.0)	12.6 (2.2)	0.15
			On Control	7.3 (1.9)	9.2 (2.0)	0.03
			Off HIBC	9.1 (1.5)	11.2 (1.7)	0.15
			Off Control	8.0 (1.6)	9.6 (1.8)	0.23
	Non-motor symptoms	PFS	HIBC	3.2 (0.4)	2.8 (0.3)	0.03
			Control	2.7 (0.4)	2.8 (0.3)	0.21
Kwok et al., 2019 [46]	Motor symptoms	MDS-UPDRS III	YP Control	34.9 (14.9) 31.6 (15.6)	22.4 (11.3) 23.3 (12.8)	0.002
		TUG	YP Control	17.5 (16.0) 14.1 (6.0)	12.4 (6.4) 13.5 (16.4)	0.99
	Non-motor symptoms	HADS-anxiety	YP Control	6.3 (3.6) 5.7 (4.0)	3.0 (3.1) 5.0 (3.5)	<0.001
		HADS-depression	YP Control	6.7 (3.4) 6.2 (3.6)	3.5 (2.8) 6.0 (3.7)	<0.001
van der Kolk et al., 2019 [44]	Motor symptoms	MDS-UPDRS III	On AIG	19.4 (1.8)	21.2 (2.0)	0.002
			On Control	17.4 (1.8)	20.3 (2.0)	
		MDS-UPDRS III	Off AIG	29.5 (2.7)	29.0 (2.5)	0.26
			Off Control	27.2 (2.7)	31.4 (2.5)	
		MDS-UPDRS IV	On AIG	2.7 (0.6)	3.3 (0.9)	0.94
			On Control	3.1 (0.6)	3.6 (0.9)	
		MBEST	AIG	24.3 (0.6)	24.4 (0.6)	0.94
			Control	24.2 (0.)	24.5 (0.6)	
		TUG	AIG	8.3 (0.5)	8.2 (0.5)	0.49
			Control	8.7 (0.5)	8.6 (0.5)	
		6MWT	AIG	499.4 (18.2)	510.6 (17.7)	0.62
			Control	486.4 (18.2)	492.8 (17.7)	
		Pegboard	AIG	19.5 (0.9)	18.8 (0.7)	0.44
			Control	19.6 (0.9)	19.4 (0.7)	
		Finger tapping	AIG	65.8 (6.4)	65.7 (6.4)	0.54
			Control	72.6 (6.4)	73.3 (6.4)	
	Non-motor symptoms	MoCA	AIG	26.3 (0.4)	25.7 (0.5) *	0.70
			Control	26·3 (0.4)	25.9 (0.5) *	
		HADS	Depression score AIG	4.2 (0.5)	4.5 (0.6) *	0.55
			Depression score Control	3.6 (0.5)	4.2 (0.6) *	
			Anxiety score AIG	4.2 (0.6)	4.1 (0.5) *	0.74
			Anxiety score Control	5.2 (0.6)	4.2 (0.5) *	
		SCOPA	AIG sleep day	3.2 (0.6)	3.5 (0.6) *	0.20
			Control sleep day	4.1 (0.6)	3.9 (0.6) *	
			AIG sleep night	4.4 (0.6)	4.6(0.6)*	0.85
			Control sleep night	4.6 (0.6)	4.6 (0.6) *	
			AIG gastrointestinal	1.6 (0.3)	1.6 (0.3) *	0.50
			Control gastrointestinal	1.6 (0.3)	1.5 (0.3) *	
		FSS	AIG	3.7 (0.2)	3.7 (0.2) *	0.52
			Control	3.9 (0.2)	3.7 (0.2) *	
		TMT	AIG Part A	39.1 (2.9)	35.5 (2.6) *	0.29
			Control Part A	40.3 (2.9)	37.9 (2.6) *	
			AIG Part B	95.0 (9.3)	83.8 (9.1) *	0.15
			Control Part B	92.2 (9.3)	90.6 (9.1) *	
		TAPF	AIG	−3.5 (2.2)	−3.6 (1.9) *	0.71
			Control	−4.4 (2.2)	−5.6 (1.9) *	
Cheung et al., 2018 [34]	Motor symptoms	UPDRS III	HY	25.6 (6.9)	17 (1.7) *	^+^
			Control	24.4 (7.3)	22.5 (1.8) *	
	Non-motor symptoms	MoCA	HY	26.9 (2.2)	28.1 (0.4)	NA
			Control	26.1 (2.4)	27.5 (0.4)	
		BDI	HY	8.8 (5.9)	8.9 (1.1)	NA
			Control	7.1 (5.0)	8.6 (1.2)	
		PDSS	HY	112.3 (22.2)	112.2 (4.1)	NA
			Control	107.2 (23.2)	106.3 (4.3)	
Son et al., 2018 [30]	Motor symptoms	6MWT	MMBCEP	373.96 (70.03)	438.68 (60.32)	<0.001
			Control	373.44 (59.47)	378.46 (59.47)	
		Shoulder suppleness	MMBCEP	124.93 (18.7)	137.71 (14.12)	0.01
			Control	129.51 (12.3)	129.24 (11.81)	
		Chair stand test	MMBCEP	12.72 (4.69)	15.23 (4.00)	0.01
			Control	13.61 (4.60)	12.95 (4.18)	
		2.45 m walk test	MMBCEP	6.94 (1.87)	5.17 (1.67)	<0.001
			Control	7.86 (1.92)	7.45 (1.92)	
	Non-motor symptoms	GDS	MMBCEP	14.25 (7.53)	10.85 (6.41)	<0.001
			Control	17.25 (7.07)	16.24 (6.07)	
		STAI	MMBCEP State	40.37 (4.77)	38.14 (9.33)	<0.001
			Control State	40.73 (8.67)	46.78 (7.79)	
			MMBCEP Trait	42.63 (5.33)	38.23 (10.26)	0.01
			Control Trait	41.74 (7.42)	45.15 (9.06)	
		MoCA	MMBCEP	22.87 (2.54)	25.86 (3.17)	<0.001
			Control	21.48 (5.13)	21.44 (5.13)	
		PDSS	MMBCEP	16.13 (8.17)	10.14 (4.90)	0.002
			Control	12.63 (6.19)	14.15 (5.09)	
Tollár et al., 2018 [40]	Motor symptoms	MDS-UPDRS M-EDL	HIAP	19.3 (5.5)	12.0 (3.7)	<0.001
			Control	18.9 (7.9)	18.6 (7.6)	
		TUG	HIAP	16.1 (3.7)	9.9 (2.7)	<0.001
			Control	18.6 (4.2)	18.2 (4.0)	
	Non-motor symptoms	BDI	HIAP	17.0 (5.3)	13.9 (5.0)	<0.001
			Control	18.0 (10.6)	17.7 (9.8)	
Collett et al., 2016 [42]	Motor symptoms	MDS-UPDRS III	EG	16.7 (10.1)	17.7 (1.1)	^+^
			Control	19.9 (9.9)	19.2 (1.2)	
		2MWT	EG	146.6 (23.9)	144.6 (2.5)	NA
			Control	137.7 (22.9)	137.9 (2.6)	
		TUG	EG	9.4 (2.0)	10.1 (0.3)	NA
			Control	10.1 (2.1)	10.6 (0.3)	
		Nine-hole peg test	EG	24.9 (5.4)	26.2 (0.6)	NA
			Control	26.8 (5.9)	25.7 (0.6)	
	Non-motor symptoms	N-MSQ	EG	8.4 (5.0)	8.9 (0.4)	NA
			Control	8.6 (4.2)	8.0 (0.4)	
		FSS	EG	3.6 (1.4)	3.6 (0.1)	NA
			Control	3.9 (1.4)	3.4 (0.2)	
Ni et al., 2016 [35]	NA					
Ni et al., 2016 [36]	Motor symptoms	MDS-UPDRS III	PWT	32.9 (12.0)	NA	0.72
			Control	27.6 (7.8)	NA	
	Non-motor symptoms	MMSE	PWT	29.1 (0.9)	NA	0.88
			Control	29.4 (1.1)	NA	
Volpe et al., 2013 [41]	Motor symptoms	UPDRS III	ID	24.58 (3.87)	17.42 (3.85)	<0.001
			Control	23.92 (3.50)	21.00 (3.07)	
		TUG	ID	NA	NA	0.007
			Control	NA	NA	
		BBS	ID	36.08 (9.20)	46.08 (6.75)	0.051
			Control	34.08 (9.14)	38.92 (9.97)	
		FOG	ID	11.42 (2.78)	4.92 (2.07)	0.001
			Control	10.75 (3.39)	10.16 (4.47)	
	Non-motor symptoms	MMSE	ID	26.5(1.4)	NA	NA
			Control	26.3(1.8)	NA	
Schenkman et al., 2012 [37]	Motor symptoms	UPDRS III	FBF	35.5 (13.9)	23.7 (1.7)	0.72 *
			AE	34.6 (13.0)	21.9 (1.8)	
			Control	37.5 (13.7)	24.2 (1.8)	
		UPDRS Total	FBF	35.5 (13.9)	32.6 (2.4)	0.62 *
			AE	34.6 (13.0)	31.4 (2.4)	
			Control	37.5 (13.7)	35.6 (2.4)	
		FRT	FBF	12.9 (3)	13.6 (0.5)	0.46
			AE	13.6 (3.1)	13.8 (0.5)	
			Control	12.5 (3.1)	13.4 (0.5)	
		CS-PFP	FBF	48.9 (17.2)	52.9 (2.4)	0.221
			AE	49.6 (15.4)	50.5 (2.4)	
			Control	44.6 (15.9)	49.6 (2.4)	
	Non-motor symptoms	UPDRS I	FBF	9.4 (4.9)	7.6 (0.8)	0.54 *
			AE	8.5 (4.8)	7.8 (0.8)	
			Control	9.6 (4.8)	9.5 (0.8)	
		MMSE	FBF	28.8 (1.1)	28.8 (1.1)	0.21
			AE	28.3 (1.8)	28.3 (1.8)	
			Control	28.8 (1.5)	28.8 (1.5)	

Abbreviations: 2MWT: 2-Minute Walk Test; 6MWT: 6-Minute Walk Test; AE: supervised aerobic exercise; AIG: aerobic intervention group; BBS: Berg Balance Scale; BD: Brazilian dance; BDI: Beck Depression Inventory; CS-PFP: Continuous-Scale Physical Functional Performance Test; DWE: deep-water exercise; EG: experimental group; FBF: supervised flexibility/balance/function exercise; FES-I: Falls Efficacy Scale—International; FFABQ: Fear of Falling Avoidance Behavior Questionnaire; FG: freeweight and elastic band group; FOG: Freezing of Gait Questionnaire; FRT: Functional Reach Test; FSS: Fatigue Severity Scale; GDS: Geriatric Depression Scale; GG: gym group; HADS: Hospital Anxiety and Depression Scale; HIAP: high intensity agility program; HIBC: high-intensity multimodal exercise boot camp; HY: Hatha yoga; ID: Irish dance; IPAQ: Physical Activity Questionnaire; MBEST: Mini-Balance Evaluation Systems Test; MDS-UPDRS: revised Movement Disorder Society Unified Parkinson’s Disease Rating Scale; MMBCEP: Mindfulness Meditation-Based Complex Exercise Program; MMSE: Mini Mental State Examination; MoCA: Montreal Cognitive Assessment; NA: not available; NMSQ: Parkinson’s Disease Non-Motor Symptom Questionnaire; NW: Nordic walking; PDSS: Parkinson’s Disease Sleep Scale; PFS: Parkinson Fatigue Scale; PWT: power-based resistance training; SCOPA: Scales for Outcomes in Parkinson’s Disease; STAI: State-Trait Anxiety Inventory; STS: Sit-to-Stand Test; TAPF: Test of Attentional Performance Flexibility; TMT: Trail Making Test; TUG: Timed Up and Go; TUG-FS: Timed Up and Go (fast speed); TUG-SSS: Timed Up and Go (self-selected speed); UPDRS I: Unified Parkinson’s Disease Rating Scale part I (non-motor experiences of daily living); UPDRS II: Unified Parkinson’s Disease Rating Scale part II (motor experiences of daily living); UPDRS III: Unified Parkinson’s Disease Rating Scale part III (motor examination); UPDRS IV: Unified Parkinson’s Disease Rating Scale part III (motor complications); UPDRS Total: Unified Parkinson’s Disease Rating Scale total score, M-EDL: motor experiences of daily living. * The *p*-value was only present in the baseline results. ^+^ *p*-value not reported, Confidence Interval/Effect Size suggestive of positive results

## Data Availability

Data will be made available upon request (t.khoo@griffith.edu.au).
